# Clinical features and outcomes of extramedullary myeloid sarcoma in the United States: analysis using a national data set

**DOI:** 10.1038/bcj.2017.79

**Published:** 2017-08-25

**Authors:** G Goyal, A C Bartley, M M Patnaik, M R Litzow, A Al-Kali, R S Go

**Affiliations:** 1 Division of Hematology, Mayo Clinic, Rochester, MN 55905, USA; 2 Division of Biomedical Statistics and Informatics, Mayo Clinic, Rochester, MN 55905, USA

Extramedullary myeloid sarcoma (MS, also known as granulocytic sarcoma, myeloblastoma or chloroma) is a rare form (<1%) of extramedullary acute myeloid leukemia (AML) presenting without bone marrow involvement.^1^ Due to the rarity of this disease, contemporary clinical data are mostly limited to small case series.^2, 3, 4, 5^ The prognostic factors and outcomes of extramedullary MS are unclear. Similar to AML, the current National Comprehensive Cancer Network guidelines recommend initial treatment with induction chemotherapy for all patients diagnosed with extramedullary MS.^6^ In this study, we utilized the National Cancer Database (NCDB) to describe the clinical features, prognostic factors and early treatment outcomes in extramedullary MS.

We identified patients with a histologically confirmed diagnosis of extramedullary MS from 2004 to 2013 using International Classification of Diseases for Oncology version 3 (ICD-O-3) code 9930 from the NCDB. The NCDB is a joint program of the Commission on Cancer of the American College of Surgeons and the American Cancer Society that collects outcomes data for over 1500 Commission-accredited cancer programs in the nation, and accounts for >70% of new cancer diagnoses.^7^

Primary site codes were used to group extramedullary MS patients by location. We excluded patients whose primary site code was bone marrow. We analyzed overall survival (OS) using Kaplan–Meier estimates and their differences between race and sex using log-rank tests. To allow for ⩾1 year of follow-up, we excluded patients diagnosed in 2013 from OS analysis. To ascertain accuracy of follow-up, the OS analysis also excluded patients whose treatment decisions were made at a facility outside of the reporting facility. Multivariate analysis was performed adjusting for age, sex, race and disease location. Because NCDB records only the initial treatment and not subsequent therapies, we performed a landmark OS analysis among those patients who survived ⩾1 month according to treatments received during the first month after diagnosis. We investigated the effect of early chemotherapy, surgery and radiation on survival using Cox regression models. This analysis was done separately for patients older and younger than 70-years of age.

A total of 94 185 cases of AML were reported from 2004 to 2013. During this time period, there were 746 patients diagnosed with extramedullary MS and comprised 0.8% of all AML diagnoses. The median age was 59 years (range, 41–73), and 56.1% were male. The distribution by race was 80.2% White, 9.1% Black, 2.9% Asian, and 7.8% others/unknown. Primary site was divided into 11 categories based on organ involved at presentation of extramedullary MS (Table 1). The three most common sites of presentation were connective/soft tissues (31.3%), skin/breast (12.3%) and digestive system (10.3%). The median OS for the entire cohort was 12.8 months. According to OS, we categorized the patients into three prognostic groups: good (OS >30 months: reproductive and digestive systems), intermediate (OS 15–30 months: head/neck, skin/breast and kidney/bladder/retroperitoneum/adrenal) and poor (OS <15 months: nervous system, connective/soft tissue, lymph nodes/spleen, cardiac/mediastinal and bones/joints). Compared to other races, Blacks were more likely to have extramedullary MS involving lymph nodes/spleen (4.4 vs 13.1%, *P*=0.03). Asians were more likely to have cardiopulmonary/mediastinal disease as compared to other races (13.6 vs 4.0%, *P*=0.02). Although both age groups had a similar proportion of poor prognosis sites (60.0%), younger (<70 years) patients were more likely to have good prognosis sites as compared to older patients (⩾70 years; 19.0 vs 10.9%, *P*=0.005). On multivariate analysis, factors affecting OS were age (*P*<0.0001), sex (*P*=0.0195), race (*P*=0.0009), as well as disease location (*P*=0.0027). Median OS was worse among males (HR 1.26; 95% CI 1.03–1.54), elderly (HR 2.42; 1.97–2.97), Blacks (HR 1.66; 95% CI 1.22–2.23) and those involving poor prognosis sites (HR 1.44; 95% CI 1.03–2.06) as compared to their counterparts.

We included 533 patients in the landmark analysis based on early treatment. Chemotherapy was administered to 37.7% of the patients, while 25.3% had surgery or radiation therapy only. The remaining (37%) patients did not undergo any treatment within 30 days of diagnosis. A very small proportion of patients underwent a hematopoietic stem cell transplant (3.4%), although the exact nature of conditioning regimen and donor source was unavailable. Early chemotherapy had no effect on OS in younger patients (HR=0.93; 95% CI=0.68–1.28). Among older patients, receiving early chemotherapy was associated with higher mortality (HR=2.59; 95% CI=1.54–4.36) (Figures 1a and b). It is worthwhile to note that a substantial proportion (19.9%) of patients underwent chemotherapy after 30 days of diagnosis (Supplementary Figure S1). Of this latter group, 25% underwent surgery and 14% underwent radiation therapy before receiving systemic chemotherapy. However, analyzing OS at later landmarks (60 and 90 days) yielded similar results for survival between chemotherapy and no chemotherapy subgroups.

Ours is the largest study of extramedullary MS in the United States. A previous study included 345 patients with extramedullary MS from the surveillance, epidemiology and end results (SEER) database from 1973 to 2010 and demonstrated superior median OS for extramedullary MS as compared to AML without MS (8 vs 5 months).^8^ This study also showed improved survival for pelvic/genitourinary, gastrointestinal mucosa, gonads/eye as compared to nervous system, soft tissue and lymph node/hematopoietic tissue. In contrast, a study from Toronto evaluated 90 patients with extramedullary MS and did not find any correlation between the site of disease and outcomes.^9^ The reason for the difference in OS among various disease sites is unclear. The initial treatment received was similar among the sites involved (Table 1). Reproductive and digestive system MS may have a better prognosis due to early symptom onset and detection, while poorer survival in bone MS may indicate early onset marrow involvement. Upon reviewing these findings, one may hypothesize that extramedullary MS involving the poor prognosis sites may benefit from early chemotherapy.

Previous institutional studies have shown that the median time to develop AML from extramedullary MS ranged from 5 to 12 months. Hence most experts recommend treating extramedullary MS with induction chemotherapy.^10^ A case series of 15 patients with extramedullary MS and 46 AML patients with MS showed that 87% (13/15) of extramedullary MS cases developed AML.^2^ The entire cohort consisted of a higher proportion of poor prognosis sites (bone and lymph node) than ours. In a subsequent series of 16 patients with extramedullary MS, only 7 (44%) developed AML.^11^ Six out of these seven patients died within 5 weeks to 16 months after diagnosis, despite systemic chemotherapy in five of those six patients. The disease sites for patients who progressed were nervous system (2), skin/subcutaneous tissue (2) and one each of uterine, ileal and nasal fossa.^11^ It is to be noted that four of the nine patients who did not progress to AML (intermediate/good prognosis sites) were alive without evidence of disease at 3.5–16 years of initial presentation and had received systemic chemotherapy.^11^ These findings, along with our study, suggest prognostic differences based on site of involvement of extramedullary MS, and median OS for some of the disease sites like reproductive and digestive system is perhaps better than AML in general.

We showed a disparity in OS according to sex and race. As seen in prior studies of AML, Blacks had a worse survival as compared to Whites.^12^ Both these differences in sex and race were not seen in prior MS series, likely due to smaller sample size.^8^ The racial disparity in survival could be ascribed to higher frequency of the poor prognosis sites (lymph nodes/spleen) in Blacks as shown in our study, or due to disease heterogeneity.

In our study, only 37% of the patients received systemic chemotherapy within the first 30 days. A similar proportion of patients did not receive any treatment during the same time period, with no difference in outcomes as compared to early systemic or local therapies in younger patients. Of note, NCDB does not contain information whether any of these patients received second line chemotherapy. Hence, our focus was on the role of early treatment. In our study, early systemic chemotherapy was associated with worse outcomes in older patients (>70 years) as compared to early radiation/surgery or no treatment. This may be explained by a lower frequency of good prognosis sites in older patients, thereby suffering from the combined impact of worse disease biology and chemotherapy-related toxicities.

The primary limitations of our study are those related to its derivation from a database. It does not give us information about the patients who progressed to AML and the exact treatment regimens received. Nevertheless, it is the largest database for malignant diseases in the United States and is especially helpful in assessing prognosis in rare diseases like extramedullary MS, where traditional clinical studies are limited by sample size.

Our study demonstrates that extramedullary MS has a diverse anatomic clinical presentation and the OS varied significantly according to age, sex, race and sites of presentation. Randomized trials analyzing optimal treatment strategy for extramedullary MS would be challenging to conduct due the rarity of the condition. Hence the results of our study may aid the prognostication of patients for treatment planning and in the understanding of the biological differences by anatomic sites of presentation.

## Figures and Tables

**Figure 1:**
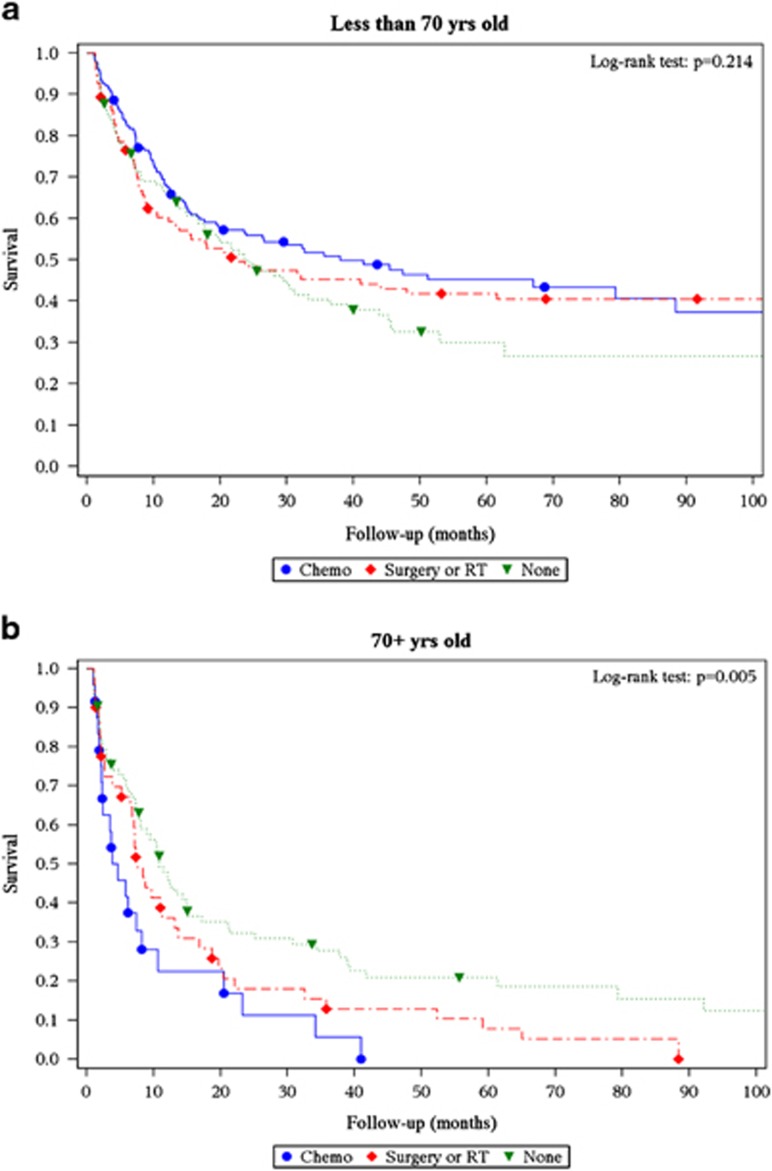
Thirty-day landmark analysis depicting overall survival based on treatment modality in patients with extramedullary myeloid sarcoma (**a**) age <70 years, and (**b**) age ⩾70 years.

**Table 1:** Anatomical distribution, survival and therapies for extramedullary myeloid sarcoma

*Anatomic location*	*All patients*			*Landmark analysis*			
	n*(%)*	*Median age*	*Median survival (months)*	n	*Chemotherapy within 30 days,*n*(%)*	*Surgery or radiation within 30 days (no chemotherapy),*n*(%)*	*No treatment within 30 days,*n*(%)*
Connective and soft tissue	234 (31.3)	61.5	10.1	152	60 (39.5)	37 (24.3)	55 (36.2)
Nervous system	46 (6.2)	33.0	10.7	34	12 (35.3)	15 (44.1)	7 (20.6)
Digestive system	77 (10.3)	55.0	32.3	59	18 (30.5)	20 (33.9)	21 (35.6)
Bones and joints	49 (6.6)	61.0	7.3	43	16 (37.2)	9 (20.9)	18 (41.9)
Head and neck	40 (5.4)	61.5	29.6	30	12 (40)	8 (26.7)	10 (33.3)
Skin and breast	92 (12.3)	65.0	19.7	69	15 (21.7)	16 (23.2)	38 (55.1)
Lymph nodes and spleen	73 (9.8)	65.0	11.6	51	24 (47.1)	6 (11.8)	21 (41.2)
Reproductive organs	43 (5.8)	51.0	88.4	36	13 (36.1)	16 (44.4)	7 (19.4)
Cardiopulmonary and mediastinum	32 (4.3)	50.5	11.1	21	12 (57.1)	4 (19)	5 (23.8)
Kidney/bladder/adrenal/retroperitoneum	37 (4.9)	62.0	28.2	24	13 (54.2)	4 (16.7)	7 (29.2)
Unknown/ill-defined	23 (3.1)	67.0	5.7	14	6 (42.9)	0	8 (57.1)
Total	746	59.0	12.8	533	201 (37.7)	135 (25.3)	197 (37)
