# Supplementary figures and images for: Clinical features and outcomes of extramedullary myeloid sarcoma in the United States: analysis using a national data set
Source: Blood Cancer J. 2017 Aug 25;7(8):e592–. doi: 10.1038/bcj.2017.79 (PMC5596389; doi:10.1038/bcj.2017.79)

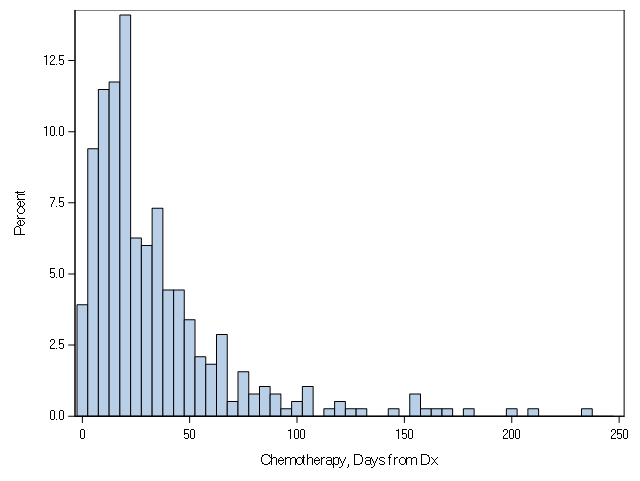


Supplementary Figure S1. Time to receipt of chemotherapy from the day of diagnosis (Dx).

Supplement: Supplementary Figure S1 [file bcj201779x1.docx]
